# Exercise-Induced Urticaria: A Rare Case Report

**DOI:** 10.7759/cureus.23062

**Published:** 2022-03-11

**Authors:** Nikita Sijapati, Monica Sciturro, Matthew Le, Jesus Lanza, Edgar Mercado, Admir Seferovic

**Affiliations:** 1 Family Medicine, St. Petersburg General Hospital, St. Petersburg, USA; 2 Family Medicine, Nova Southeastern University Dr. Kiran C. Patel College of Osteopathic Medicine, Clearwater, USA; 3 Intensive Care, St. Petersburg General Hospital, St. Petersburg, USA; 4 Critical Care, St. Petersburg General Hospital, St. Petersburg, USA

**Keywords:** exercise-challenge-test, anaphylaxis, urticaria, allergy, exercise

## Abstract

Exercise is an important part of a healthy lifestyle. However, there is a subset of the population who are allergic to exercise. Exercise-induced urticaria is a rare clinical condition, which, as the name suggests, manifests as flushing, pruritus, and hives following physical exercise. A minority of patients even develop more severe reactions including angioedema and anaphylaxis induced by exercise. Some patients are affected by certain cofactors that constitute food-dependent exercise-induced urticaria, which is relatively more common when compared to exercise-induced urticaria without other cofactors. This case report documents a healthy 27-year-old Asian male, with no other allergies or cofactors, who was diagnosed with exercise-induced urticaria. He was diagnosed based on history and a positive exercise challenge test. Avoidance of exercise is the mainstay of prophylactic treatment for this condition. Modification of physical activity proved to be effective for treating this patient. We intend to increase awareness about this rare condition through this case report and literature review.

## Introduction

Allergies are most commonly triggered by pollen, dander, foods, drugs, detergents, and other external agents; however, some people develop allergies as a reaction to physical exercise. This allergic reaction usually manifests within 30 minutes of starting the exercise and can range from pruritus and urticaria, which is described by the term "exercise-induced urticaria," to angioedema and respiratory distress, encapsulated by the term "exercise-induced anaphylaxis (EIA)." Most people with this condition also have cofactors such as allergies to wheat or other specific food items, which, in conjunction with exercise, triggers this response. We present a rare case report of a 27-year-old male with exercise-induced urticaria, without any triggering cofactors, who developed wheals within 20 minutes of starting the exercise.

## Case presentation

The patient was a 27-year-old Asian male, with no significant past medical history, who presented with self-reported "exercise-induced rash." He had first noticed it while playing soccer at the age of eight years. Since then, he had consistently developed a raised, erythematous rash on his arms during every soccer practice and this had limited his physical activity. At 22 years of age, the patient had begun to consistently develop generalized urticaria within 20 minutes of engaging in running. The urticaria usually self-resolved in two hours. The patient denied swelling of his lips or tongue, throat discomfort, or difficulty in breathing. He denied any new food intake, medication, cosmetic use, trauma, or insect bites prior to any episodes. He developed urticaria with physical activity regardless of food intake prior to the exercise. The patient had no known allergies to food or medication. The only surgical history was related to a wisdom tooth extraction. He denied the use of tobacco, alcohol, or recreational drugs.

**Figure 1:**
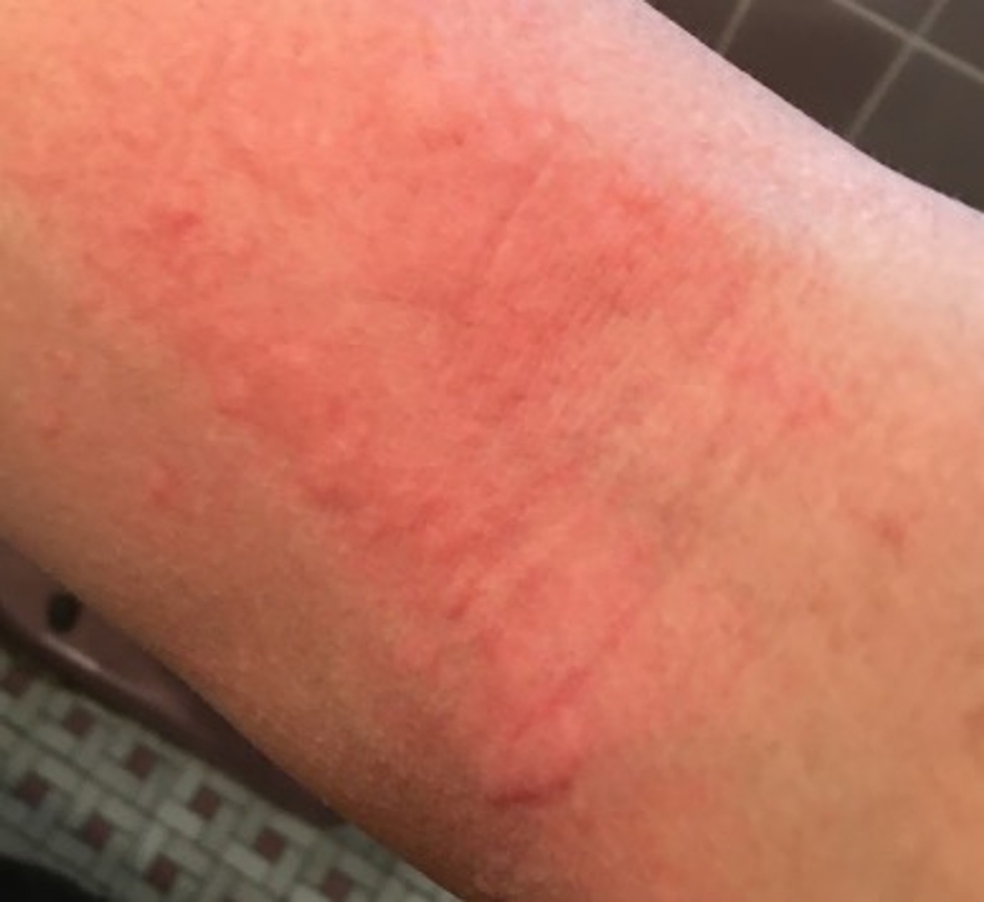
Urticaria in the volar aspect of the right arm within 20 minutes of the onset of the exercise

**Figure 2:**
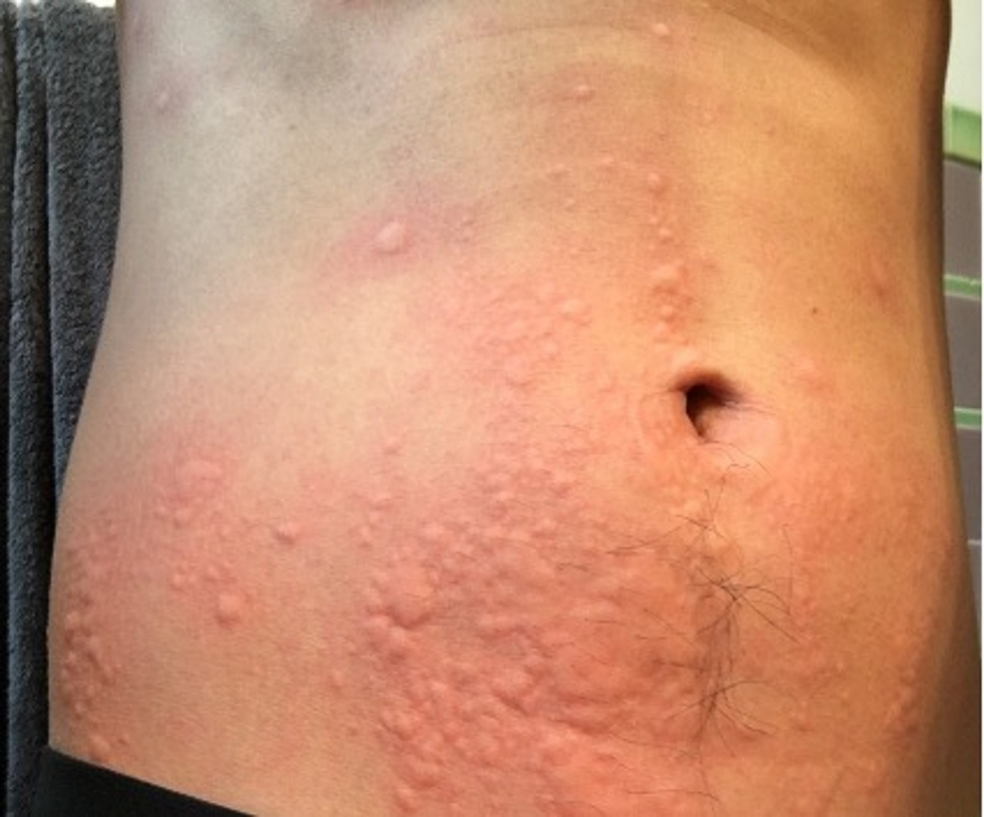
Urticaria on abdomen within 20 minutes of the onset of the exercise

**Figure 3:**
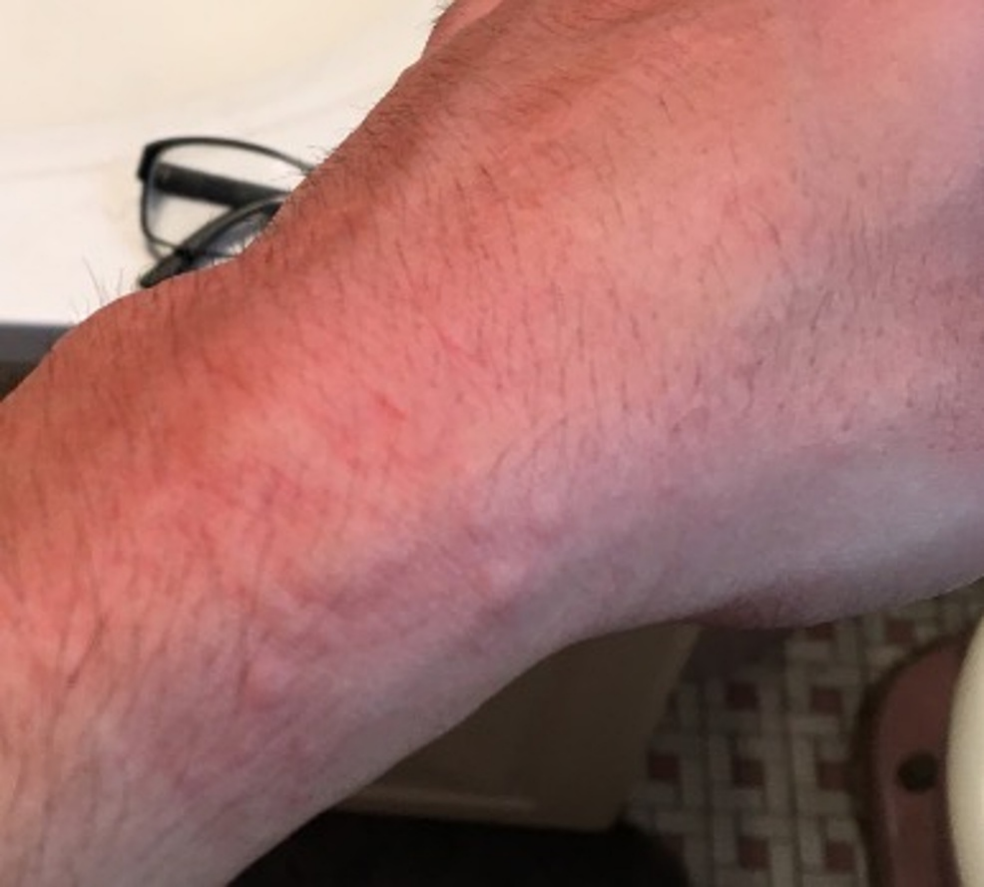
Urticaria on the left wrist within 20 minutes of the onset of the exercise

**Figure 4:**
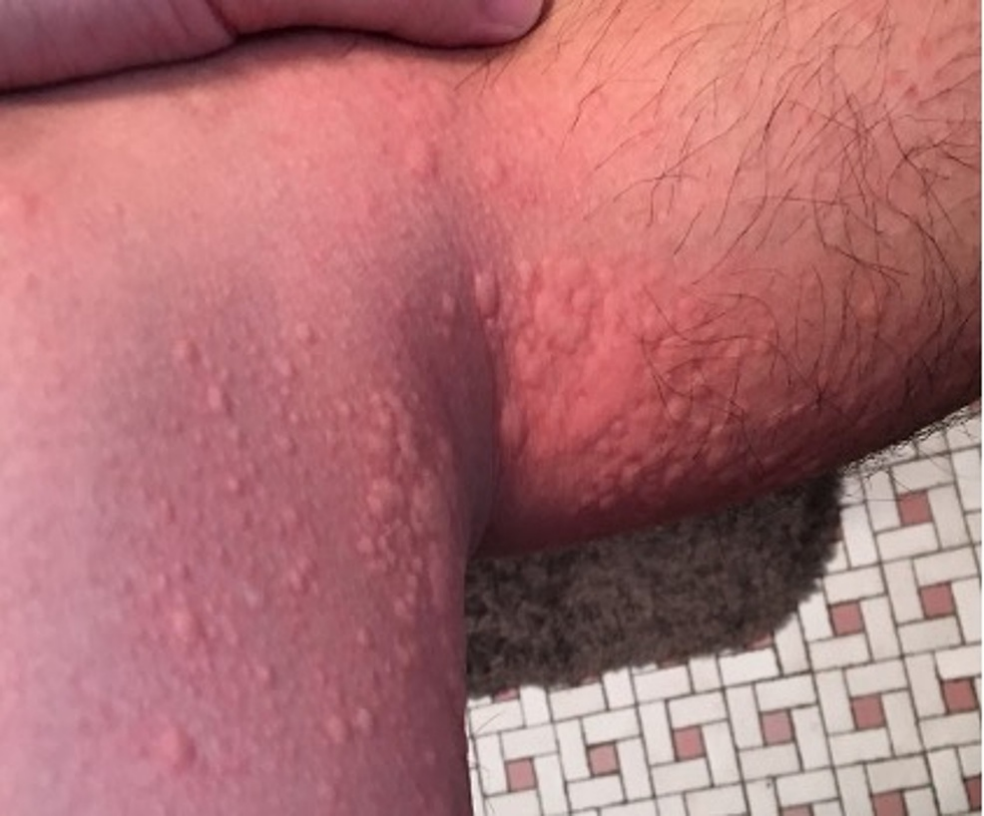
Urticaria on left axilla within 20 minutes of the onset of the exercise

In the absence of an acute episode, the patient had an unremarkable physical exam. An exercise challenge test was done in the clinical setting where the patient did a combination of running, jumping jacks, and squats. This was done under medical supervision and emergency medications were on stand-by if needed. The patient’s last food intake had been more than four hours prior. He was sweating and flushing within 10 minutes and then developed pruritus with multiple raised erythematous rashes, typical of urticaria, 17 minutes after the onset of exercise. Urticaria was noted on the volar aspects of both arms, the neck, chest, and abdomen (Figures 1-4). The patient had clear breath sounds without any wheezing and was saturating 99% on room air. There was no evidence of angioedema or anaphylaxis. Vital signs before and after the exercise challenge test showed an increase in temperature by 0.4 °C and a 30% increase in heart rate from the baseline (Table 1).

**Table 1:** Patient's vital signs pre- and post-exercise challenge

Variables	Before exercise	After exercise
Temperature	37.2 °C	37.6 °C
Heart rate	78 beats per minute	102 beats per minute
Blood pressure	115/72 mmHg	128/85 mmHg
Respiratory rate	16 breaths per minute	20 breaths per minute

The patient was diagnosed with exercise-induced urticaria based on history and a positive exercise challenge test. No additional investigation was done. He was counseled about treatment options, including avoidance of exercise or taking prophylactic antihistamines before light exercise should the patient choose to continue with some form of exercise. Rather than absolute avoidance of exercise, the patient elected to switch to low-intensity exercise through shared decision-making. The patient was advised, on initial and follow-up visits, to use oral antihistamines before every exercise session; however, he preferred not to take the medication. With the gradual modification of exercise over a period of eight months, the patient is now able to tolerate walking for an hour and swimming without triggering any allergic symptoms.

## Discussion

Exercise-induced urticaria was first described in the 1970s and continues to be a relatively rare condition. Its clinical presentation includes pruritic, white or erythematous, nonpitting edematous plaques associated with exercise, mostly jogging or running, which may progress to anaphylaxis [1]. The lesions are likely the result of an increased body temperature that leads to capillary vasodilation and transudation of fluid into the superficial dermis. Three different types of exercise-induced urticaria have been described: cholinergic urticaria, classic EIA, and variant EIA [1,2]. The morphologic size of the urticarial wheals can help distinguish between these conditions. Cholinergic urticaria is induced by exercise, body warming, and emotional stress, and clinically manifests as small papules that are surrounded by an erythematous halo [2]. Lesions can spread to the entire body but normally start around the thorax and neck [2,3]. The variant form presents similarly to cholinergic urticaria but is triggered only by exercise [3]. The classic form of EIA is usually accompanied by upper respiratory obstruction and hypotension [4].

Although the pathophysiology of exercise-induced urticaria and anaphylaxis is not well understood, thermoregulatory mechanisms and an exaggerated cholinergic response may have a role [5]. An increase in temperature of more than 0.7 °C, mast cell degranulation, and increase in plasma histamine level have been proposed to be responsible for the symptoms [5,6].

The diagnosis is usually based on history but an exercise challenge test or a methacholine skin test may be conducted. However, the methacholine test is only positive in one-third of patients, and hence a negative result does not rule out a diagnosis [3,7]. The patient in this case report was diagnosed based on typical history and a positive exercise challenge test. Plasma histamine and serum immunoglobulin levels are not ordered routinely. These laboratory studies were offered to our patient, but he declined to undergo them, as it would not impact our management.

The prophylactic treatment of exercise-induced urticaria involves the avoidance of exercise [1,8]. However, given the benefits of exercise on overall health, modification of physical activities can be attempted after discussing with patients. Antihistamines such as loratadine, fexofenadine, and hydroxyzine may be effective to a certain extent in preventing symptoms [1,9]. Mast cell stabilizers and leukotriene-modifying agents have been described, but more studies are required to establish their effectiveness [1]. Avoiding food for at least four to six hours prior to exercise has been mentioned across the literature as a way to help prevent food-dependent exercise-induced urticaria [1,7,8]. Although most literature mentions avoidance of exercise and antihistamines as the mainstay of treatment, for our patient, avoidance of exercise initially was followed by gradual switching to low-intensity exercise and swimming. He was able to tolerate low-intensity exercise without the use of antihistamines. This case report indicates that a gradual modification of exercise can be an effective treatment modality if individualized to the patient's tolerance levels and severity of symptoms. Further research is required to establish a more structured algorithm for exercise modification among these patients.

## Conclusions

Exercise-induced urticaria is a rare medical condition characterized by symptoms of pruritus and wheals during or shortly after physical exercise. It can be diagnosed based on history, an exercise challenge test, or a methacholine skin test. Its pathophysiology is still not fully understood. The typical treatment involves avoidance of exercise; however, modification of exercise can also be attempted in mild cases. Avoiding food right before and after exercise, use of antihistamines before exercise, and use of mast cell stabilizers have been studied with variable results. It is important for family physicians, who are advocates of a healthy lifestyle and exercise, to be aware of this rare condition as well as know how best to counsel patients to prevent it from occurring.
